# Supplementary material for: Deglycosylated Azithromycin Attenuates Bleomycin-Induced Pulmonary Fibrosis via the TGF-β1 Signaling Pathway
Source: Molecules. 2021 May 10;26(9):2820. doi: 10.3390/molecules26092820 (PMC8126120; doi:10.3390/molecules26092820)
Supplement: Supplementary file 1 [file molecules-26-02820-s001.zip › molecules-1185415-supplementary.pdf]

Supplementary Material

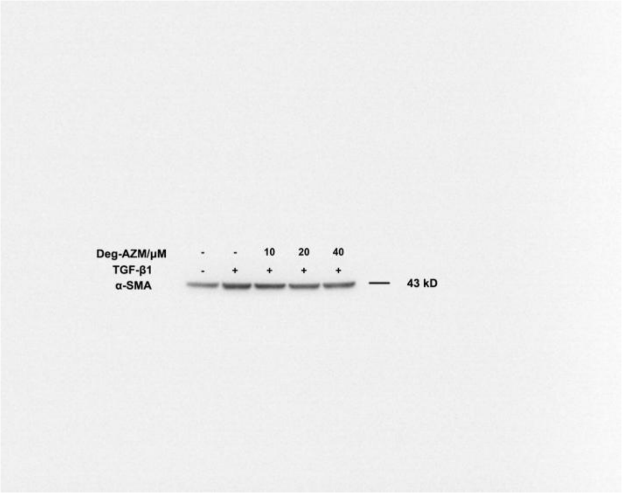

Supplementary Figure 1. The entire original gel of α-SMA in Figure 5D.

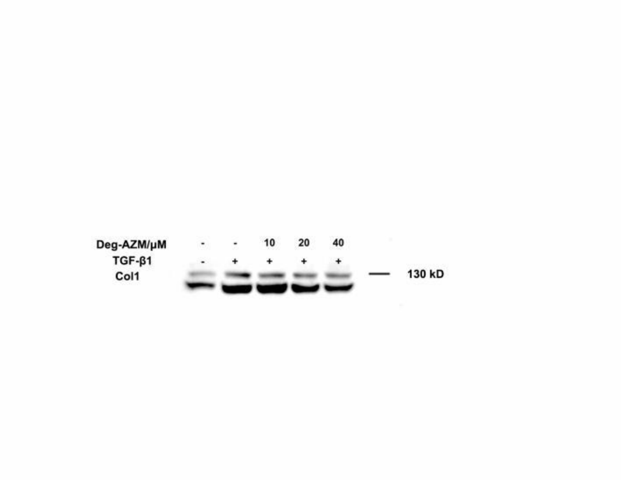

Supplementary Figure 2. The entire original gel of Col1 in Figure 5D.

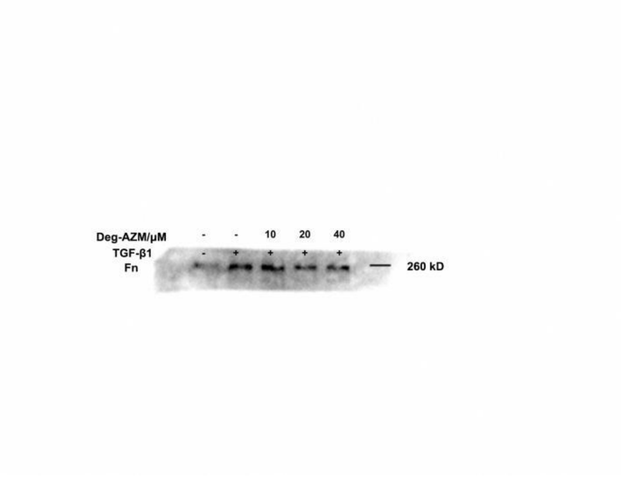

Supplementary Figure 3. The entire original gel of Fn in Figure 5D.

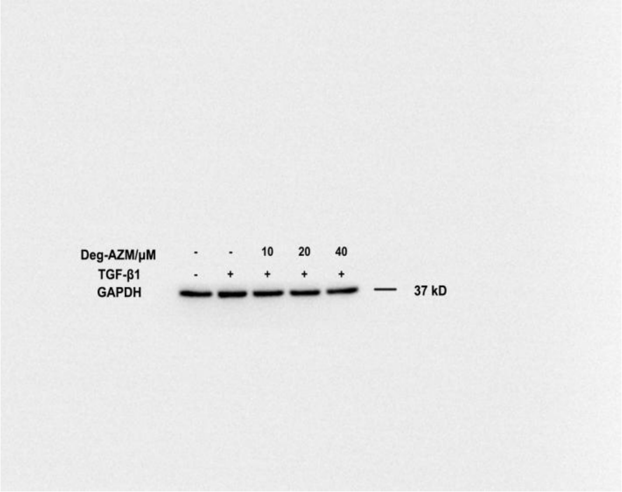

Supplementary Figure 4. The entire original gel of GAPDH in Figure 5D.

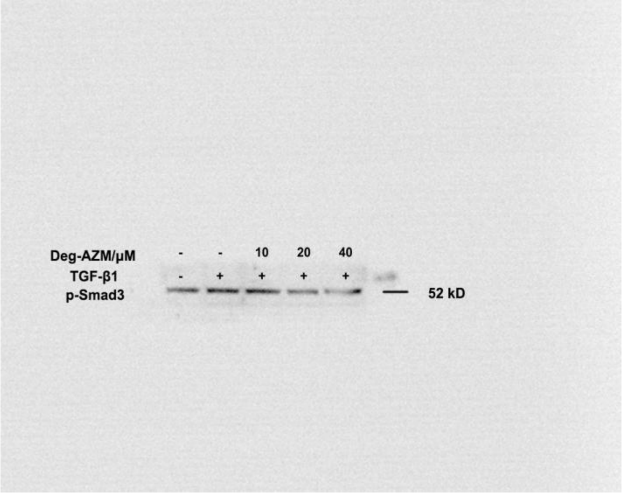

Supplementary Figure 5. The entire original gel of p-Smad3 in Figure 5I.

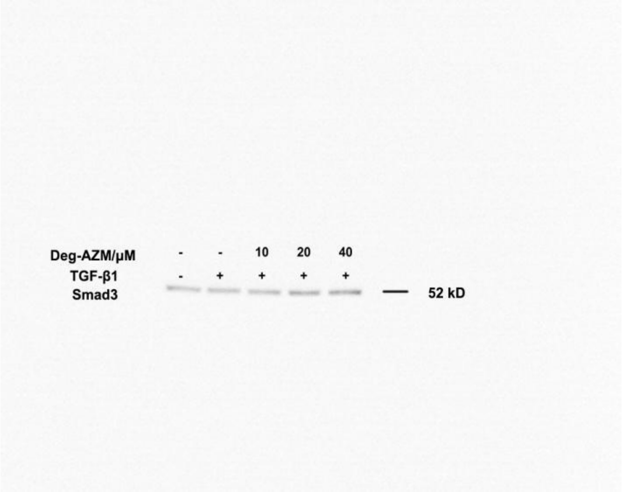

Supplementary Figure 6. The entire original gel of Smad3 in Figure 5I.

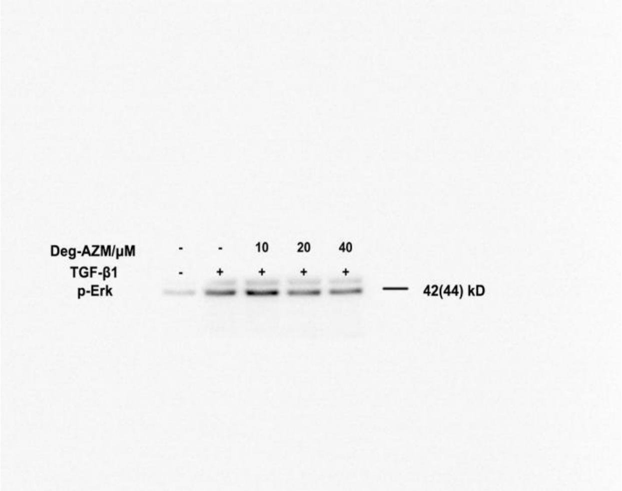

Supplementary Figure 7. The entire original gel of p-Erk in Figure 5I.

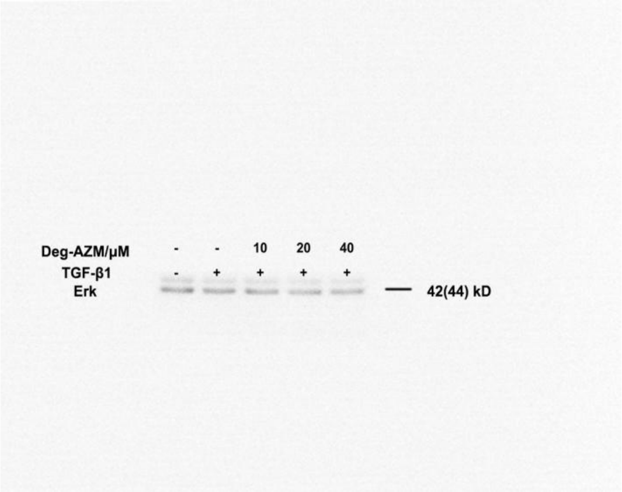

Supplementary Figure 8. The entire original gel of Erk in Figure 5I.

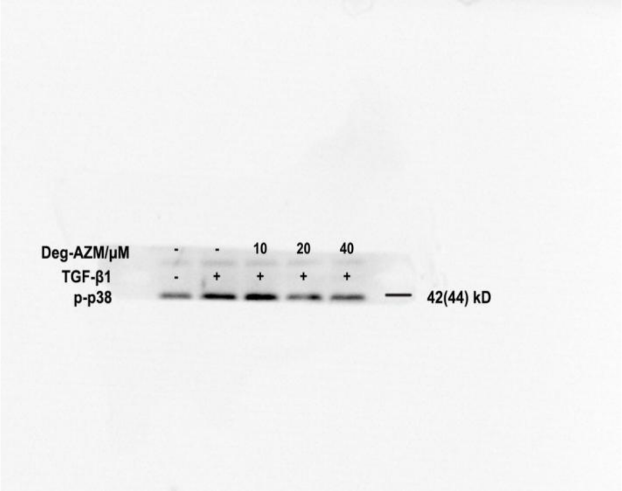

Supplementary Figure 9. The entire original gel of p-p38 in Figure 5I.

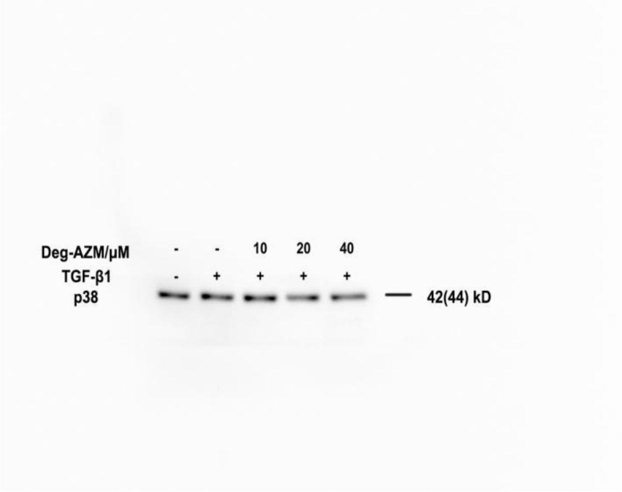

Supplementary Figure 10. The entire original gel of p38 in Figure 5I.

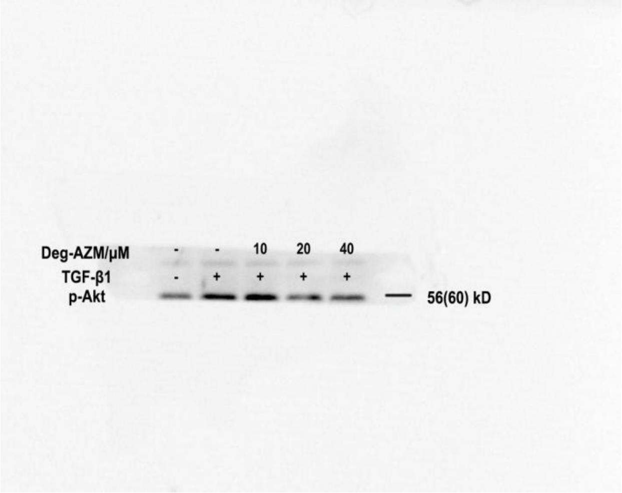

Supplementary Figure 11. The entire original gel of p-Akt in Figure 5I.

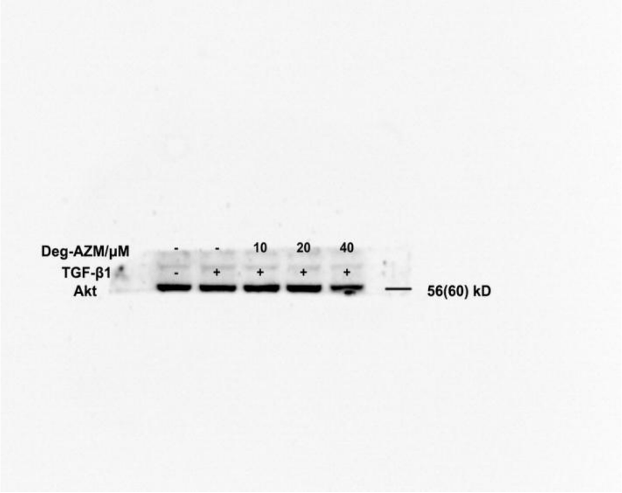

Supplementary Figure 12. The entire original gel of Akt in Figure 5I.

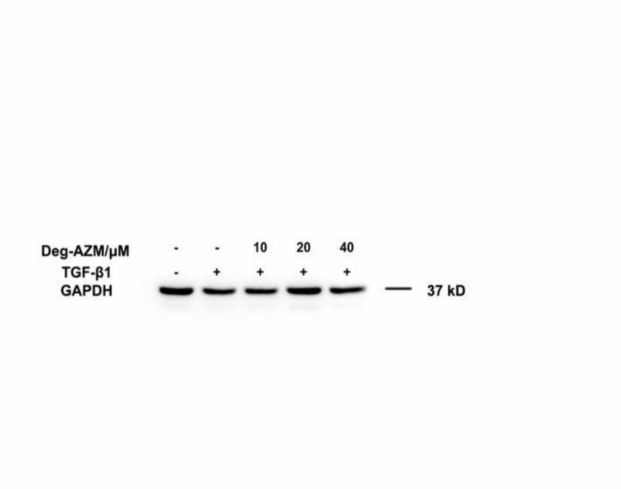

Supplementary Figure 13. The entire original gel of GAPDH in Figure 5I.

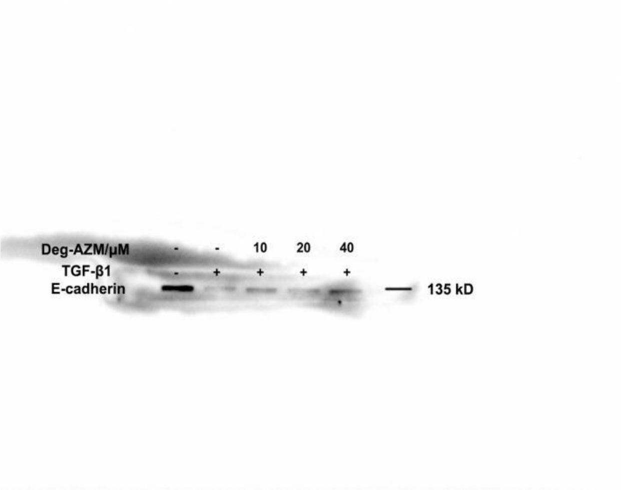

Supplementary Figure 14. The entire original gel of E-cadherin in Figure 6D.

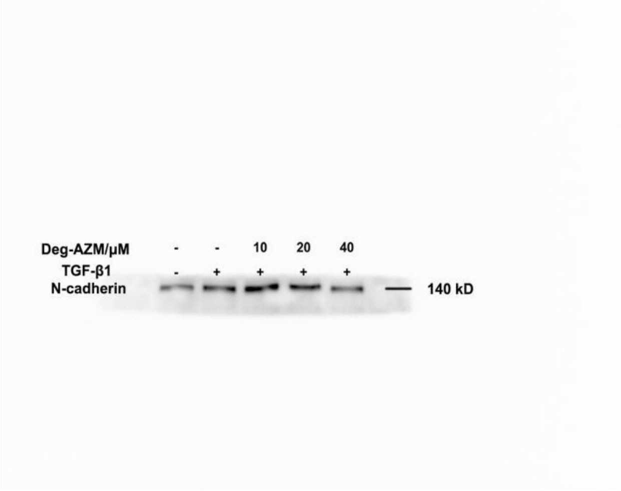

Supplementary Figure 15. The entire original gel of N-cadherin in Figure 6D.

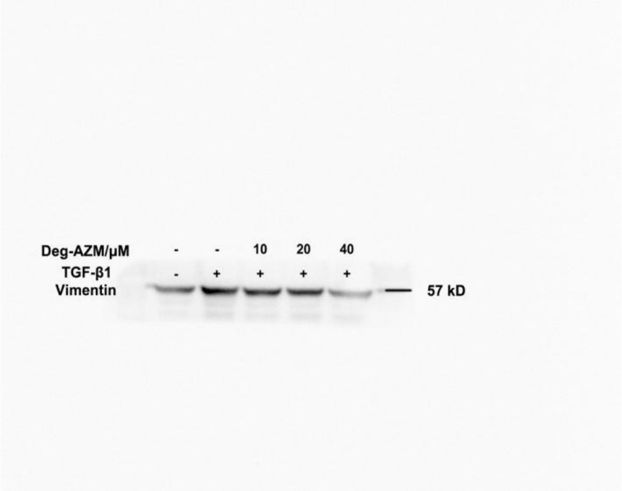

Supplementary Figure 16. The entire original gel of Vimentin in Figure 6D.

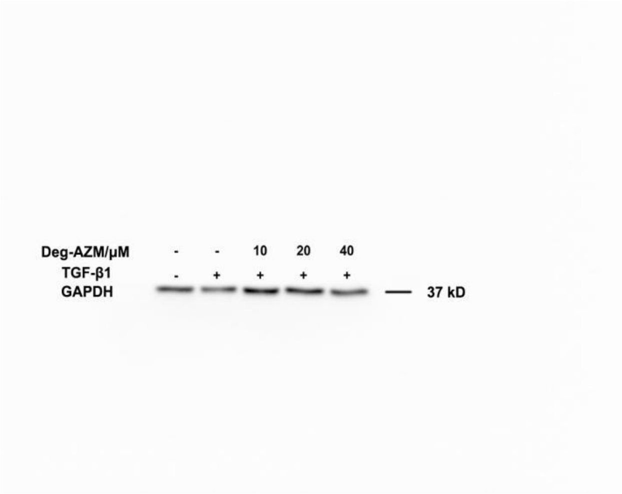

Supplementary Figure 17. The entire original gel of GAPDH in Figure 6D.

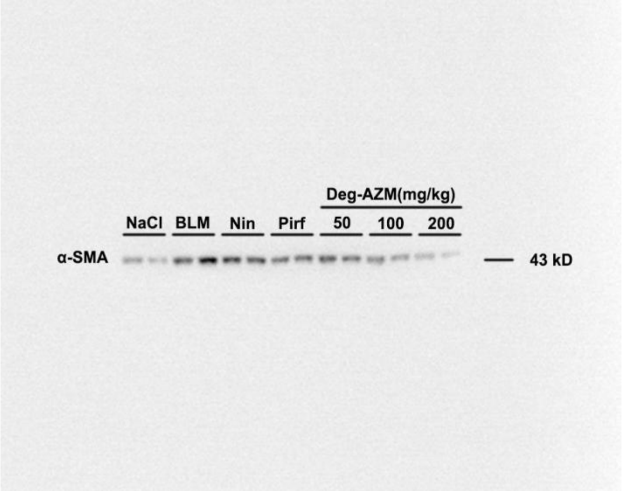

Supplementary Figure 18. The entire original gel of α-SMA in Figure 7A.

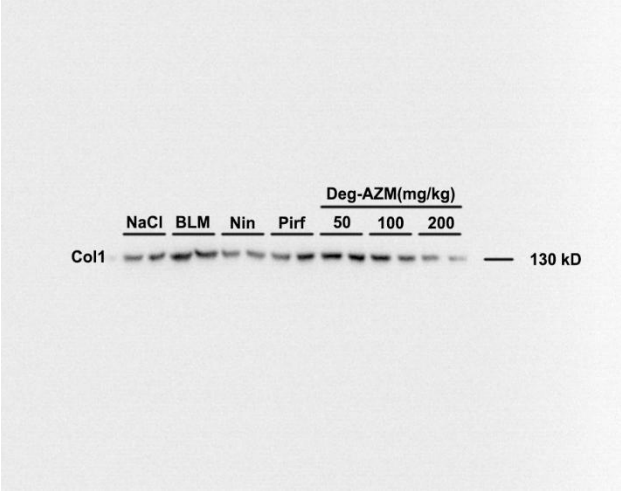

Supplementary Figure 19. The entire original gel of Col1 in Figure 7A.

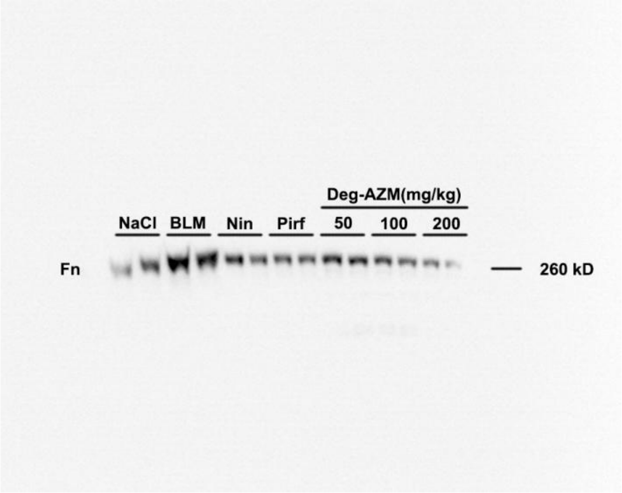

Supplementary Figure 20. The entire original gel of Fn in Figure 7A.

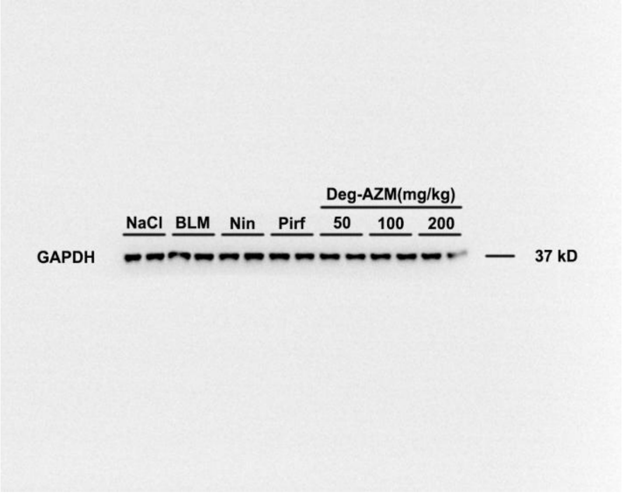

Supplementary Figure 21. The entire original gel of GAPDH in Figure 7A.

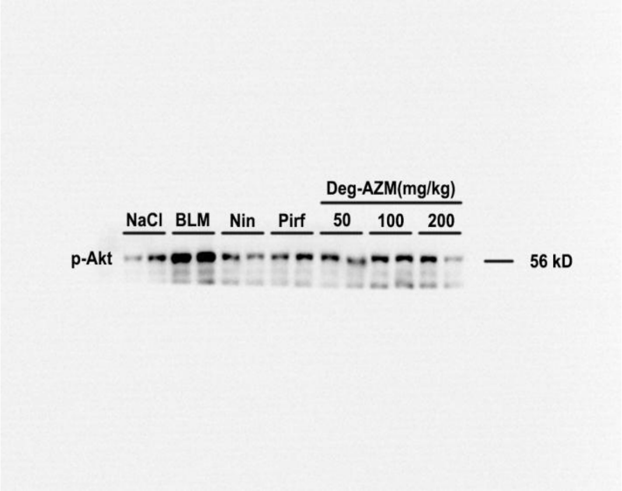

Supplementary Figure 22. The entire original gel of p-Akt in Figure 7K.

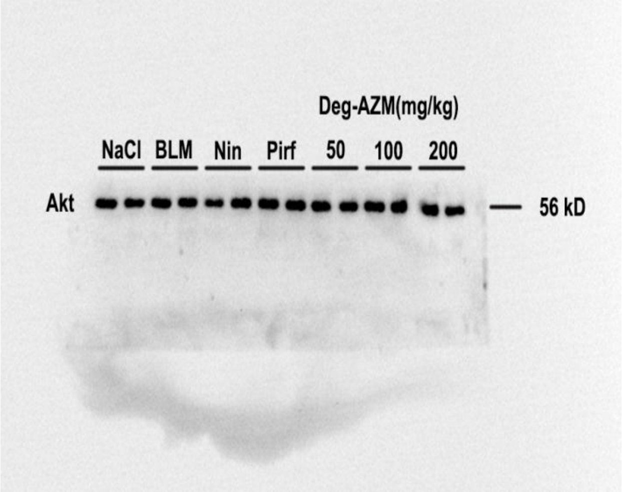

Supplementary Figure 23. The entire original gel of Akt in Figure 7K.

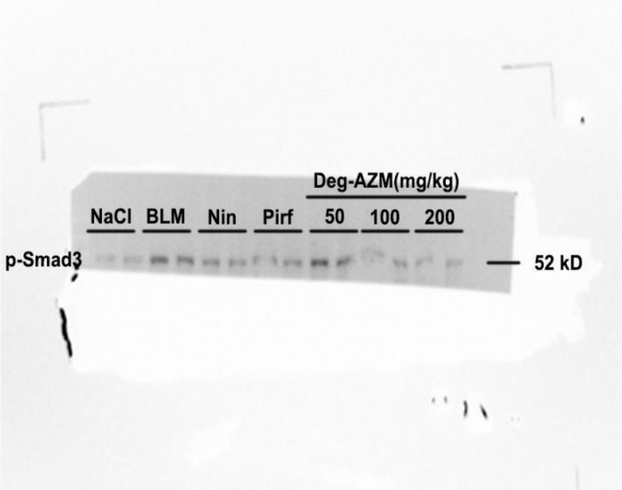

Supplementary Figure 24. The entire original gel of p-Smad3 in Figure 7K.

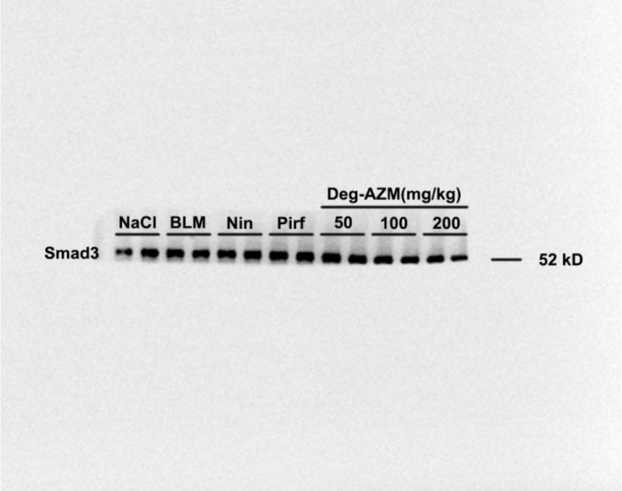

Supplementary Figure 25. The entire original gel of Smad3 in Figure 7K.

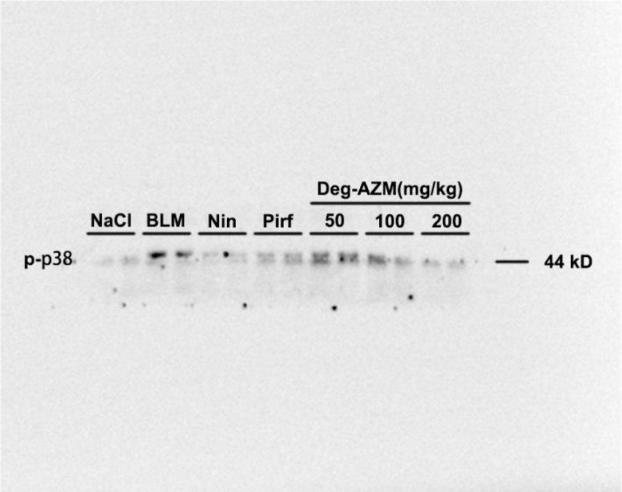

Supplementary Figure 26. The entire original gel of p-p38 in Figure 7K.

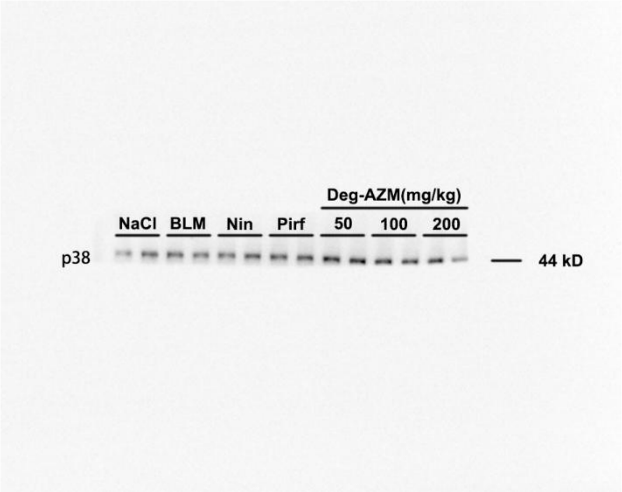

Supplementary Figure 27. The entire original gel of p38 in Figure 7K.

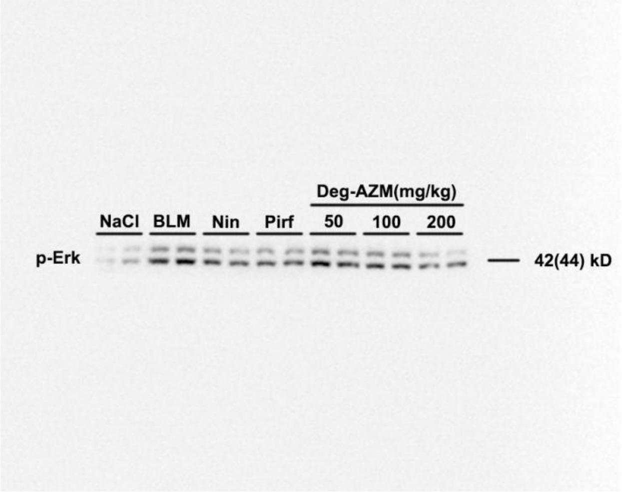

Supplementary Figure 28. The entire original gel of p-Erk in Figure 7K.

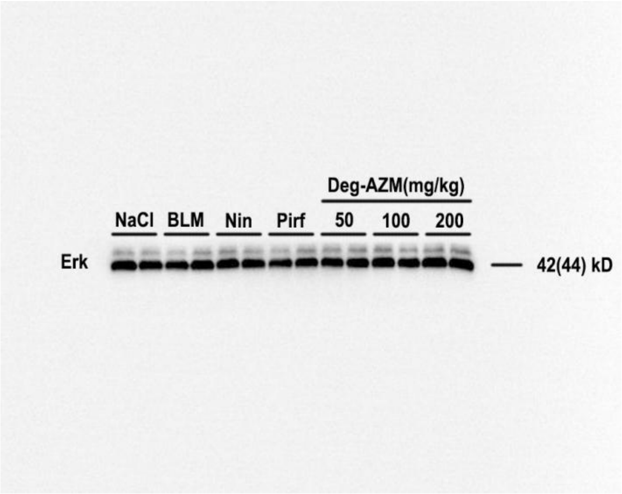

Supplementary Figure 29. The entire original gel of Erk in Figure 7K.

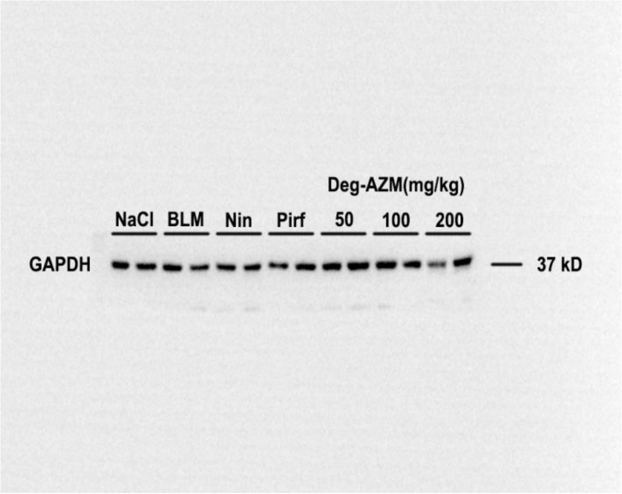

Supplementary Figure 30. The entire original gel of GAPDH in Figure 7K.

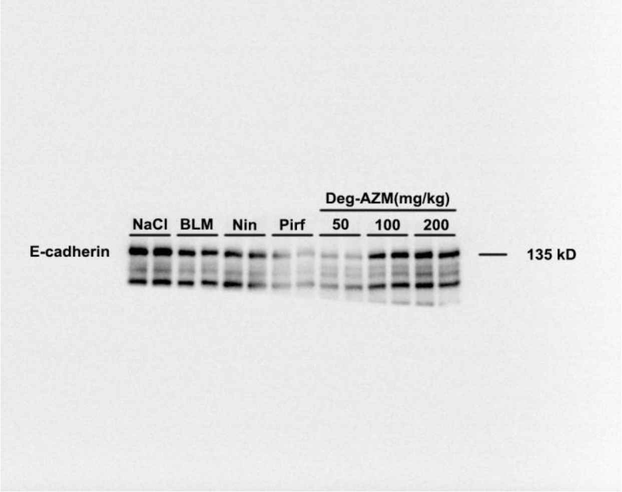

Supplementary Figure 31. The entire original gel of E-cadherin in Figure 8A.

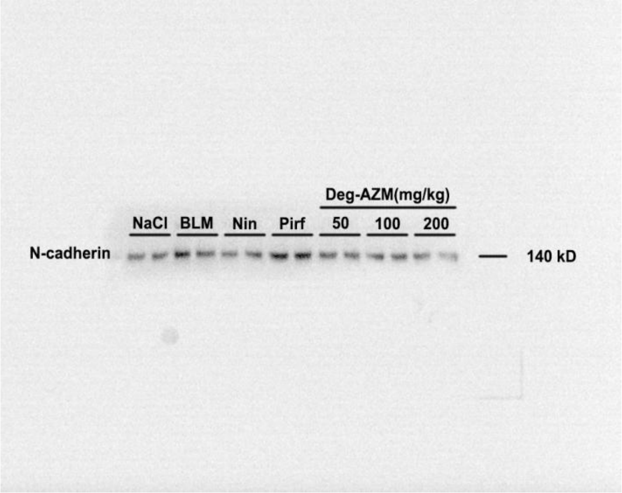

Supplementary Figure 32. The entire original gel of N-cadherin in Figure 8A.

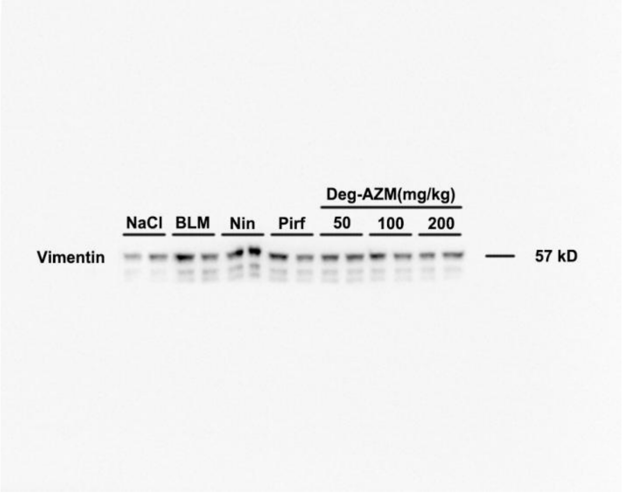

Supplementary Figure 33. The entire original gel of Vimentin in Figure 8A.

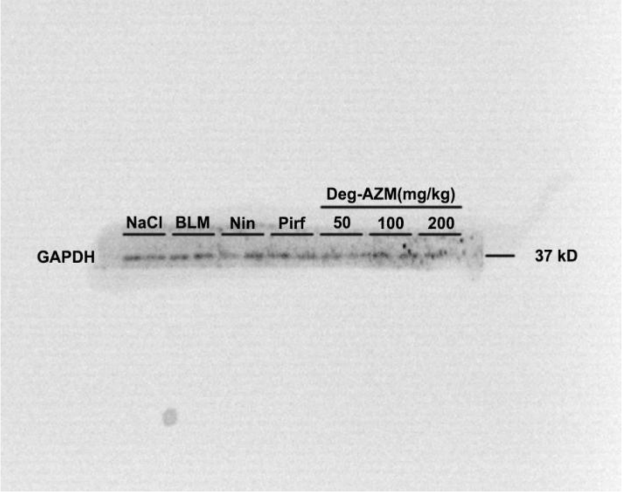

Supplementary Figure 34. The entire original gel of GAPDH in Figure 8A.
